# Development and Effect of Virtual Reality Practice Program for Improving Practical Competency of Caregivers Specializing in Dementia

**DOI:** 10.3390/healthcare9101390

**Published:** 2021-10-18

**Authors:** Dooree Kim

**Affiliations:** College of Nursing, Konyang University, Daejeon 35365, Korea; kdr2015@konyang.ac.kr; Tel.: +82-42-600-8570

**Keywords:** dementia, caregiver, virtual reality, practice, competency

## Abstract

The number of dementia patients in Korea is increasing with the increase in the elderly population. Accordingly, the importance of the role of the caregivers, who are the main care worker other than the family, is increasing. Therefore, in this study, a virtual reality practice program was developed to enhance the practical competency of caregivers who take care of dementia patients, and the effects were analyzed. The caregiver said that among the mental behaviors of dementia patients, aggression and delusion were the most difficult. Based on this information, a practice program was developed by realizing a case of a male dementia patient who expressed refusal to bathing help as an aggressive behavior due to delusion in virtual reality, and the effect of the virtual reality practice program was analyzed for five caregivers. As a result, ‘interest in new teaching methods’, ‘improving concentration of practical education based on real cases’, and ‘increasing confidence in caring for dementia patients’ were found. As this study is a pilot test, it is necessary to repeat the study with more subjects in the future, and to develop virtual reality implementation cases for various mental and behavioral symptoms.

## 1. Introduction

### 1.1. Background

According to the report of the third comprehensive dementia management plan in Korea, the rapid increase in the number of dementia patients due to aging in Korea is unprecedented in the world [1]. In 2026, the elderly population is expected to enter a super-aged society, where the elderly will make up more than 20% of the total population. The prevalence of dementia will double every 17 years, reaching approximately 1 million in 2024 and 2.71 million in 2050. It is predicted to increase to 15% of the elderly [2]. It is estimated that one dementia patient occurs every 12 min, with an annual rate of 7.9 per 1000 people aged 65 and over. Along with this increase in the elderly population, the prevalence of dementia is accelerating, and accordingly, interest in caregivers, who are key practical personnel for daily living assistance for dementia patients, is increasing. In 2008, Korea provided long-term care benefits to support physical activity or housekeeping activities to the elderly who have difficulty performing daily life alone for more than 6 months, due to old age or geriatric diseases, etc. The long-term care insurance system for the elderly was introduced to improve the quality of life of these people by alleviating the burden on the family [3]. Accordingly, the number of elderly care facilities nationwide, which was 3312 as of 2010, has reached 21,290 nationwide as of 2018 [4]. Furthermore, the number of elderly people with dementia aged 65 or older who received a rating to use the long-term care insurance system for the elderly increased from 4500 in 2010 to 9211 in 2018 [4]. In addition, the number of caregivers who provide care to those closest to them increased from 206,344 in 2015 to 273,613 in 2018, accounting for the highest ratio compared to medical personnel, such as nurses, doctors, and physical therapists [5]. Therefore, a caregiver’s role is very important in the health management of people with dementia who receive long-term care insurance services. In addition, on 1 July 2014, the role of caregivers was further emphasized for dementia patients, as long-term care for the elderly ‘dementia special grade (grade 5)’ was newly established to provide long-term care services for the increasing number of patients with mild dementia. The dementia special grade is for the elderly (including patients with dementia under the age of 65) who have difficulty in performing daily life due to cognitive impairment and problem behavior among patients with mild dementia who have been unable to receive long-term care services due to their relatively good physical functioning [6]. Accordingly, caregivers will provide cognitive activity-type programs to recipients of special dementia grades, such as recall training and memory enhancement activities to prevent cognitive deterioration and maintain residual ability 3 times a week after completing specialized dementia education [6]. The caregiver qualification acquisition process was introduced on 1 July 2008, and on 26 April 2010 it was changed to the national examination system. The caregiver training course started to strengthen the skills and knowledge level compared to home service workers and life guidance workers, which are considered as manpower under the Elderly Welfare Act [7]. If you look at the current training curriculum for new caregivers, you can take the national exam after completing 80 h each of theory, practice, and field practice for a total of 240 h [8]. However, it is judged that a total of 240 h is insufficient to learn the contents of physical, emotional, and practical care of subjects with various geriatric diseases as well as dementia. This can also be seen from the results of the evaluation of educational programs targeting caregivers in previous studies, showing that in-depth education on dementia diseases, nursing, and end-of-life care is necessary [9]. In addition, as a result of surveying dementia-related knowledge among caregivers in a previous study that looked back to about 10 years ago in the training course for caregivers, the correct answer rate for all questions was 66.4%, and the error rate of ‘the cause of dementia symptoms and problem behavior’ question was very high [10]. In addition, the education requirement for dementia was relatively high at 121 points out of 160, and the items with the highest demand included methods of coping with dementia patients’ violent behavior, wandering, refusal to receive care, sleep disorders, delirium, delusions, etc. In other previous studies, caregivers answered that coping with problem behaviors occurring while caring for dementia patients was the most difficult in practice, and they expressed the need for practical education [11]. As such, caregivers are complaining of various difficulties in caring for dementia patients. This may later reduce job satisfaction and further reduce the quality of services of caring for dementia patients [9]. For this reason, practical training for caregivers that take care of dementia patients is required, but there is a limit to carrying out practical training for patients before they have obtained a certificate for personal information protection and other various reasons. Therefore, practical education through virtual reality that embodies real situations will help caregivers to take care of actual dementia patients after obtaining their certificate. In addition, since virtual reality enables repetitive learning, the effectiveness of practical education can be enhanced.

Therefore, in this study, we intend to prepare basic data for the development of practical training programs for the quality of care for dementia patients by caregivers, by analyzing the current caregiver training curriculum and by examining and analyzing the demand for realistic, necessary education for caregivers who currently take care of dementia patients in practice.

### 1.2. Study Purpose

The purpose of this study is to develop a virtual reality practice program to enhance the practical competency of caregivers who take care of dementia patients, and to investigate the effect of the program. The specific objectives are as follows.

- Analyze the current curriculum of caregivers and analyze the actual educational needs through interviews.
- Develop cases for improving the practical competency of caregivers in caring for dementia patients.
- Implement virtual reality (VR) cases for improving the practical competency of caregivers in caring for dementia patients.
- Conduct a pilot test to evaluate the effectiveness of a virtual reality (VR) practice program developed to enhance the practical competency of caregivers in caring for dementia patients.

## 2. Study Method

### 2.1. Study Design

This study is a methodological study to develop a virtual reality practice program for improving the practical competence of caregivers in taking care of dementia patients.

### 2.2. Study Process

In this study, a virtual reality practical program was developed and evaluated by analysis, design, and development, and the pilot test was based on ADDIE (analysis, design, development, implementation, evaluation) model among teaching cases (ISD: instructional system development) to systematically develop a virtual reality practice program for caregivers taking care of dementia patients.

### 2.3. Virtual Reality Practice Program Development and Evaluation

#### 2.3.1. Analysis

##### Analysis of the Current Situation: Analysis of Domestic Caregivers’ Training Courses

In this study, in order to analyze the current caregiver training curriculum in Korea, the caregiver training curriculum, caregiver qualifications examination, and job competency improvement curriculum were analyzed, which are published on the website of the Korean Nursing Care Association (http://www.silvercare.org/; accessed on 20 November 2020).

##### Target Needs Analysis: Investigation of Educational Needs for Caregivers

1. Study subjects

The subject of this study, a caregiver, is a person who has completed a prescribed training course at a caregiver training institution and has passed the examination conducted by the National Examination Institute for Health and Medical Services and obtained a qualification issued by the governor. They are also paid employees who are paid by the facility. The subjects of this study were caregivers who participated in the dementia specialist training course conducted by the National Health Insurance Service in D city in November 2019. The selection criteria for this study were those who had experience in caring for dementia patients and those who fully understood and voluntarily agreed to the purpose of this study. For this study, the researcher used the lunch break of the curriculum to explain the purpose and method of this study, and distributed questionnaires to 85 subjects who voluntarily agreed to participate in the study. The consent form could be submitted separately. A final 77 questionnaires were collected.

2. Measurement

In this study, the researcher directly developed the questions to find out the educational needs of caregivers who have experience in caring for dementia patients. The developed tool consisted of 11 general characteristics (clinical experience of caring for dementia patients, educational experience in dementia, interest in dementia, experience of living with a family member with dementia, etc.) and 4 descriptive questions. Based on research conducted on caregivers so far, the descriptive question composition consists of ‘What is the most difficult part of caring for dementia patients as a current caregiver?’, ‘What is the reason?’, and ‘What do you think is the most necessary education while caring for a patient as a current caregiver?’, ‘What is the reason?’, and ‘Are there any other stories you would like to tell about caring for dementia patients?’. The developed questionnaire was evaluated for content validity by 3 nursing experts, and the CVI score was 0.97.

3. Result analysis

In this study, a content analysis method was performed to analyze the research data [12,13,14]. The content analysis process was divided into three stages: preparation, organizing, and reporting. The content analysis method was divided into inductive and deductive approaches, and in this study, it was analyzed through the inductive approach. In the preparation stage, efforts were made to understand the characteristics of the subjects of this study, and to select representative subjects from the population. For the inductive content analysis for organization, first, open coding was performed by taking notes or adding titles while reading the subjective responses of the questionnaire conducted in this study. The researcher read the material repeatedly and gave as many titles as needed, and based on this created categories freely. In addition, five research questions (‘Who is the speaker?’, ‘Where did it happen?’, ‘When did it happen?’, ‘What happened?’, and ‘Why?’) were considered when the data were analyzed. Second, after open coding, the number of categories was reduced by grouping similar or different categories once again, while grouping the list that created categories with higher-level titles. Finally, the determined categories were abstracted so that they can be interpreted in a universal sense according to the research topic.

In order to secure the accuracy and validity of this result analysis, first, the researcher tried to maintain parentheses in order to accept the contents of each subject’s statements in a neutral attitude. Second, the researcher tried to reflect the experience of the subject’s answers in the statements as much as possible in the analysis. In addition, the results of the analysis were reviewed with a professor from the nursing department with experience in qualitative research, and key words were discussed. Finally, I tried to describe in detail the data collection process, analysis process, and results of this study.

#### 2.3.2. Design

##### Learning Goals and Topic Selection

Based on the analysis of the first stage, the learning goals and topics of the practice program that can enhance the competence of dementia caregivers who actually care for dementia patients were selected.

#### 2.3.3. Development

- Practice program case development: based on the topic selected by the analysis process, a case study program was developed.
- Expert validity: contents developed as a case study program were evaluated for content validity by experts, such as nursing professors and actual clinical practitioners, and the final virtual reality practice program case was developed based on this process.
- Virtual reality implementation of practice program example: the finally developed practical program was implemented through virtual reality.

#### 2.3.4. Evaluation

In order to find out the effect of the finally developed virtual reality practice program, a practice program was conducted for five caregivers who were currently taking care of dementia patients, and an interview was conducted to find out their satisfaction.

## 3. Data Collection and Ethical Considerations

To conduct this study, the researcher received approval from the K University Bioethics Committee (KYU-2019-297-01). For data collection, the researcher directly visited the place where the monthly dementia specialized education was held at the National Health Insurance Service in D city, explained the purpose and method of this study to the caregivers who participated in the education and voluntarily participated in the study. A questionnaire was distributed to the subjects who consented. Subjects who consented to the study were asked to fill out a written consent form, and in the subject questionnaire, the purpose and duration of the study, research method, risks and benefits, confidentiality, and contact information of the researcher who could be contacted in case of question were written. It was explained that participation in this study was voluntary, and if the research subject did not want to participate in the study, it can be stopped at any time and there is no penalty for this. It was explained that the collected data would be used only for the purpose of the study, and to ensure the anonymity and confidentiality of the research participants, access to the collected data was prohibited except for the researcher, and the collected questionnaires were immediately encoded and stored. In addition, the researcher visited during lunch time so as not to interfere with the education, and for the collection of research data, the consent form and questionnaire box were placed at the exit so that they could freely submit it, and the researcher collected the box later.

## 4. Result

### 4.1. Virtual Reality Practice Program Development

#### 4.1.1. Analysis of Domestic Caregiver Training Courses

Currently, a caregiver is a person who provides high-quality care services, such as physical activity or housework to the elderly who have difficulty in performing daily life alone due to old age or geriatric diseases, etc., and has acquired a certificate from the national qualification system for caregivers. In July 2008, in preparation for the long-term care insurance system for the elderly, caregivers were newly established as a national qualification system as a caregiver in order to strengthen their skills and knowledge level compared to home service workers and life guidance workers, which are the former manpower under the Elderly Welfare Act. A total of 240 h of training must be completed in order to acquire the national qualification system for caregivers. The 240 h curriculum consists of 80 h of theoretical lectures, 80 h of practical practice, and 80 h of field practice. The theory consists of an introduction to nursing care (systems and services related to nursing care, purpose and function of nursing care work, professional ethics and attitudes of nursing care providers, understanding of recipients of care), and basic knowledge related to nursing care (basic medical and nursing knowledge). Practical practice includes nursing care specialties (basic care protection technology, housework and daily life support, communication and leisure support, service use support, care work record and report), and special care (dementia care technology, end-of-life and hospice care technology, first aid technology). Lastly, field training consists of senior care facility training and home care service practice. Table 1 shows the time allocation for theory and practice for each topic. Those who have completed the 240 h of education can take the exam four times a year, and they will take the written exam and the practical exam. The written exam has 35 questions on the theme of nursing care and the exam time is 40 min. The practical exam is about nursing care, has 45 questions, and the exam duration is 50 min. The form of the test is a written test with multiple-choice questions, and only those who score more than 60% of the full marks in the written and practical tests can acquire the certificate.

#### 4.1.2. Analysis of Educational Needs for Caregivers

##### General Characteristics of Subjects

The general characteristics of the subjects of this study were 73 (98.6%) women, which accounted for most of them, with 40 (55.6%) under 60 years of age and 32 (44.4%) over 60 years of age. As for educational background, 56 people (78.9%) had a high school diploma or higher, and 39 people (55.7%) answered that they had experience in dementia education. The number of ‘no’ was higher with 42 (62.7%) of those who had practical experience related to dementia care. In addition, 42 people (63.6%) answered that they had no experience of living with dementia patients. Lastly, the average clinical experience of caring for dementia patients of the study subjects who participated in this study was 2 years and 4 months.

##### Content Analysis

Question 1. “What is the most difficult thing about caring for dementia patients as a caregiver right now?”

**Figure 1:**
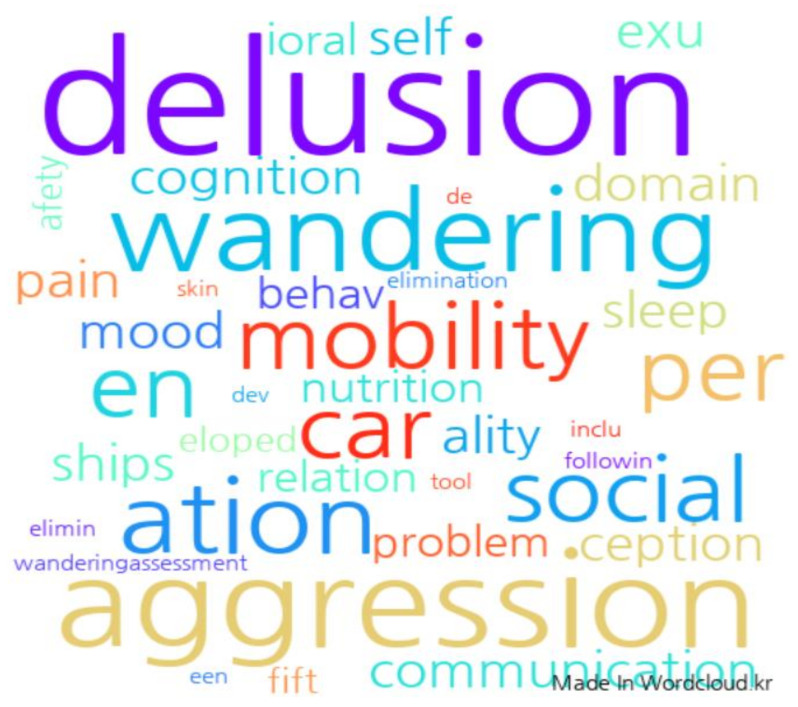
Cloud analysis of the most difficult mental behaviors of dementia patients (Wordclound.kr accessed on 3 December 2020).

In this study, the most difficult points in caring for dementia patients were ‘delusion’, ‘doubt’, ‘anxiety’, ‘agitation’, ‘aggression’, ‘hallucination’, ‘swearing and wandering’, ‘repetitive behavior’, ‘other (sexual behavior, etc.)’, and about eight items were derived, shown in Table 2. In this study, to check the frequency of the most difficult psycho-behavioral symptoms in caring for dementia in the study subjects, the answers to the questions were analyzed using a word cloud. As a result of the analysis, it was found that ‘aggression’, ‘delusion’, and ‘swearing and wandering’ were the most frequent, see Figure 1. Analyzing the responses of the subjects of this study, they said that they were mentally unstable and difficult due to the aggressive behavior of dementia patients, and that they had a sense of physical threat when providing care. In addition, in the case of the study subjects providing home care services, the subjects were more afraid of male participants because they were strong and could not be stopped. In addition, due to delusions, when providing meals while providing care, it was expressed that it was difficult to cope with the delusions, such as ‘I took medicine to die quickly’ or ‘I stole a bankbook, bag, etc.’. The subject’s swearing led to the caregiver’s feelings of being personally ignored in providing care, a fear of verbal violence, etc., and they also expressed difficulties with wandering around at the same time, or trying to go outside due to hallucinations. It was also said that when trying to restrain behaviors, such as delusions, swearing, and wandering, this became the cause of other aggressive behaviors.

Question 2. “As a caregiver, what do you think is the most necessary education for caring for dementia patients?”

In this study, the most necessary education for caring for dementia patients was classified into knowledge (theory) and practice (practice). Theory education included ‘knowledge related to dementia’, ‘understanding caregivers for dementia patients’, and ‘self-management education for caregivers with dementia’. Performance education included ‘execution of dementia prevention management program’, ‘day-to-day care for dementia patients’, and ‘how to deal with care for dementia patients’. The contents requested by the study subjects were knowledge of the dementia itself, understanding of dementia patients, symptoms and characteristics of patients at each stage of dementia, mental behavior of dementia patients, and drugs necessary for treatment of dementia patients. The study subjects providing home services also expressed the need for education of caregivers with dementia. The caregivers of dementia patients were told that they needed education on how to communicate and discuss the problems of the dementia patients with family, and other than that, the need for education on management of dementia caregivers themselves was also expressed. The study subjects seemed to feel they had many limitations while caring for dementia patients. Accordingly, the necessity of sharing information through self-help groups among people caring for dementia patients was expressed, and education on how to deal with depression and sudden anger arising from caring for patients on their own was requested, as well as how to be patient. The educational content requested for performance included that they would like to be able to directly perform cognitive stimulation programs that can be applied to actual dementia patients, activity methods tailored to the characteristics of dementia patients, and exercise programs to maintain residual functions. In caring, they expressed their hope that they would have an opportunity to learn while actually performing pressure sore management, fall management, and diaper nursing during bowel movements. In addition, it was requested that they learn through practice how to deal with emergency situations that may occur in caring for dementia patients, communication practice with dementia patients, and how to cope with mental and behavioral symptoms, see Table 3.

Question 3. “Is there anything else you would like to say about caring for dementia patients?”

In this study, the contents of the stories that the subjects of this study wanted to tell about caring for dementia patients were largely classified into ‘Needs for practical education for caring for dementia’ and ‘Needs for services for families of dementia patients’. The study subjects said that practical education for dementia care, that is, programs such as multi-faceted counseling and demand resolution of caring for dementia patients, were necessary. The necessity of time to share each other’s experiences was suggested through self-help groups for caregivers. In addition, they suggested that it would be desirable to have more educational opportunities to experience many cases in real situations. As for the improvements for dementia care, it is necessary to prepare a system so that the quality of care can be improved through maintenance education for workers in facilities twice a year, and it is necessary to provide care in consideration of the gender of the caregiver and the subject. In addition, it was suggested that it would be desirable to expand the opportunities for specialized dementia education courses that can be applied through the head facility. Lastly, the importance of education for families of dementia patients was also presented. The necessity of activating self-help groups for families of dementia patients and providing professional educational opportunities for families with dementia was suggested.

##### Design

Topic selection and learning goals

**Figure 2:**
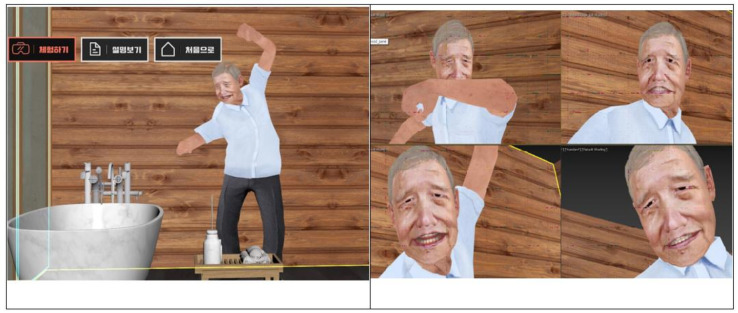
Scenario (Left picture icon description: Experience/Watch the video/For the first time).

In this study, based on the results of analyzing the educational needs of caregivers taking care of dementia patients, a case was developed under the theme of aggressive behavior among the mental behavioral symptoms of dementia patients (a case of dementia patients showing aggression when taking a bath due to the delusion of trying to kill themselves), and it was implemented in virtual reality. The case structure is shown in Figure 2. The goals of this virtual reality practice program are, first, to explain the definition of psycho-behavioral symptoms of dementia patients. Second, it is possible to understand the aggressive behavior among the psycho-behavioral symptoms of dementia patients. Third, it is possible to explain how to cope with aggressive behavior among the psycho-behavioral symptoms of dementia patients. 

##### Development

a. Case development of virtual reality practice program

The topic of the virtual reality practice program of this study was selected as ‘Understanding and coping with delusional behavior in dementia patients’, based on the result of educational requests in which ‘aggression’, ‘delusion’, ‘swearing’, and ‘wandering’ were the most reported through the experience of caring for dementia patients using word cloud, and the most difficult point in subjective answers were expressed as aggressive behavior due to delusions. Accordingly, the trainees were selected as caregivers taking care of dementia patients in the current long-term care facilities, and the place of practice was selected as the researcher’s laboratory. The virtual reality practice program in this study was a total of 60 min, 10 min of program introduction, 15 min of lecture on mental and behavioral symptoms of dementia patients, 5 min of virtual reality practice, and 10 min of mental behavior cases and coping methods of dementia patients. The program satisfaction survey consisted of 10 min, shown in Table 4.

b. Expert validity: For the content validity of the virtual reality practice program developed in this study, content validity was performed with the following subjects: one medicine professor with dementia research experience, one occupational therapy professor, two nursing professors, and one long-term care facility head with experience in nursing dementia patients. As a result, feedback on program time and gender of virtual reality characters was reflected and completed.
c. Case virtual reality implementation

To implement the case of the dementia patient’s aggressive behavior developed in this study into virtual reality, the character, facial expression, and aggressive behavior pattern of the dementia patient were selected through a meeting with a virtual reality implementation company. In addition, voices and situational backgrounds were selected through several discussions. Considering the age of the subjects of this study, the cases in which virtual reality can be experienced were composed of a total of 4 min and 30 s (Appendix A). The start of the virtual reality practice program enabled the first definition of the psycho-behavioral symptoms of dementia patients, and the psycho-behavioral symptoms to be experienced in this study were specified. After this, a grandfather with a cane appeared in the background of the bathroom for bathing, and the situation of taking the grandfather to the bathroom was realized. When a subject tried to take the grandfather to the bath, the grandfather swung his arms back and forth and swung his cane, and it was constructed to express refusal such as ‘Go away! Don’t do it!’, see Figure 2. The VR implemented in this study was developed through HTC’s Vive Pro, and at least two base stations and a vive controller were used for user tracking.

d. Evaluation of the effectiveness of Korean-style virtual reality practice program

In this study, the initial plan was to train five people as a group, but group meetings were limited due to COVID-19, so the virtual reality practice program was conducted one by one. The subjects of this study were five caregivers who had experience in caring for dementia patients through working at S Nursing Home, located in S city. Before performing the program, the subject was contacted to determine the date of participation in the program, and the subject visited the researcher’s laboratory according to the date, and the researcher explained the purpose and contents of this study and obtained written consent before starting the virtual reality practice program. Afterwards, the definition and types of psycho-behavioral symptoms of dementia patients were explained, and the introduction and precautions for virtual reality were briefly explained before performing virtual reality practice. Subjects who participated in this study had an average age of 65 years or older, and it was determined that there may be risks such as dizziness during virtual reality experience, so this study was conducted in a sitting position. After experiencing the virtual reality practice program, the study subjects shared information about similarities with their actual experiences. After that, we discussed and explained how to deal with situations, such as the case in the virtual reality practice program. Finally, the study subjects shared their experiences through this practice program. It was found that ‘Interest in new teaching methods’, ‘Improving concentration of practical education based on real cases’, and ‘Increasing confidence in caring for dementia patients’ summarized the feelings of the five subjects. The common reports from subjects were ‘To experience the patient in a very realistic way’, ‘It is amazing that it is possible to experience real work situations through virtual reality’, ‘ It was good to know the method of coping with problems that have been troubling through examples.’, etc. Furthermore, there were opinions, such as ‘I think it would be helpful if I was given this opportunity before the actual start of work’, and ‘I want to experience other psycho-behavioral symptoms as it can be helpful in understanding people with dementia.’ In addition, there were opinions such as ‘It was the first time I did it, so it was awkward to use the machine, and I was afraid because I had to cover my eyes’, ‘I felt a little dizzy’, and ‘I felt uncomfortable because I was wearing glasses’.

## 5. Discussion

This study focused on the development of a virtual reality practice program to improve the practical competency of caregivers taking care of dementia patients, and a pilot test was conducted to find out the effect. In this regard, I would like to discuss the development process and effects of the virtual reality practice program.

In this study, in order to develop a virtual reality practice program, we analyzed the curriculum for caregivers who are currently caring for dementia patients in Korea, surveyed their educational needs, and conducted content analysis. As a result, the current curriculum for obtaining the caregiver certificate in Korea is 240 h in total, consisting of 80 h each of theoretical lectures, practical practice, and field practice. Currently, practical practice involves learning theory through textbooks rather than direct technical performance, and field practice is also conducted mainly by observation, due to changes in the medical environment that puts the patient’s rights first and the complexity of the clinical environment. Opportunities for practice were limited. However, practical practice and field training for caregivers caring for dementia patients are very important processes to apply theory and knowledge to the field in practical practice to provide actual patient care.

Currently, there is no research on practical skills and field training for caregivers in Korea, so it is difficult to compare them. However, compared to the case of nursing students who have a similar curriculum and undergo hospital practice, nursing students also recently prioritized the rights of patients, and due to the changed clinical environment, the practice is focused on observation. As a result, it was found that clinical practice stress occurs due to the limitation of practical application of theory and knowledge [15]. Such clinical practice stress has been shown to have an effect on nursing professionalism and further negatively affect professional awareness and professional values [16,17]. It may have a negative effect in the sense of vocation and occupational values for the caring of people with dementia, and it is thought to be a cause of lowering the quality of care for dementia patients. In addition, as a result of analyzing the educational needs in this study, the most difficult aspects of caring for dementia patients were found to be aggression, delusions, and swearing from the dementia patients. 

Caregivers expressed that they were psychologically anxious and found it difficult because they did not know the reason and coping methods for these behaviors of dementia patients. This can be understood in the same context as in previous studies, as an influencing factor of caregiver burden of caregivers of dementia patients were shown to be psychotic symptoms, such as hallucinations and delusions, aggression, and wandering behaviors of dementia patients [18,19,20]. It can be understood as there were complaints of a high burden through caring for the elderly with dementia due to assault and verbal abuse caused by the psycho-behavioral symptoms of the elderly [21]. In addition, 95% of caregivers experienced verbal and 91% physical violence, and 21.5% experienced physical damage due to the aggressive behavior of dementia patients [22,23]. Caregivers who have these experiences undergo mental shock and helplessness, which in turn leads to chronic fatigue, loss of work motivation, and psychological disorders, which lowers the quality of care for dementia patients and further leads to resignation [24,25]. In order to reduce the burden of care for caregivers of dementia patients, Wright’s coping methods involve continuously securing appropriate information about the environment, maintaining efficient internal conditions for behavior and information processing, maintaining autonomy, and acting flexibly [20]. Therefore, in this study, a virtual reality practice program was developed to improve the understanding of the mental and behavioral symptoms of dementia patients, and to educate them on how to deal with this. Furthermore, it was intended to improve the quality of care for dementia patients. Therefore, as a result of the analysis of the subjects’ needs, a case was developed under the theme of aggression and delusion, which were the most frequent among the mental behaviors of dementia patients which were expressed to be the most difficult.

The virtual reality education program was an experience-oriented education that increases concentration and interest, and can guarantee safety through experience in virtual reality. In particular, in the recent medical field, direct contact with patients in clinical settings or performing techniques is often limited, due to safety issues for patients and students. Accordingly, education that can provide treatment situations experience through virtual reality is increasing [26,27]. Caregivers also play a role in caring for real patients in the local community, so mistakes that may occur in real situations can be prevented through case experiences, according to the virtual reality practice program before providing care to patients. In addition, even if care is currently provided, the quality of care for dementia patients can be improved through repeated learning of various cases.

The subjects of the case developed in this study were selected as male dementia patients. This is because most of the caregivers are female, but as a result of the content analysis in this study, it was found that male dementia patients felt more fear when they acted aggressively. In addition, the case was selected as a situation of refusing to take a bath due to aggressive behavior stemming from delusion, and trying to harm the caregiver. In fact, in this study, the situations in which the most aggressive behavior occurred were bathing refusal and eating refusal.

The virtual reality practice program developed in this study was provided to five caregivers who were currently caring for dementia patients. This program consisted of a total of 60 min, and due to COVID-19, the group education initially planned was changed to individual education. As a result of analyzing the contents of interviews with participants who completed the virtual reality practice program to find out their satisfaction with it, ‘Interest in new teaching methods’, ‘Improving the concentration of practical education based on real cases’, and ‘Increased confidence in taking care of actual dementia patients’ were common responses. ‘Interest in new teaching methods’ and ‘Improving the concentration of practical education based on real cases’ were results of this study, although they had heard stories about virtual reality through the mass media, and all subjects who experienced virtual reality for the first time expressed interest in the experience itself. In addition, it seems that the interest in learning increased experiential learning that goes beyond the limitations of time and space as well as other educational methods, compared to the existing educational media. In addition, the result of improved concentration compared to conventional education can be understood in the same context as the results of previous studies, in which the level of concentration in learning increased due to the sense of security that it is okay to make mistakes [28,29]. 

The following analysis result, ‘Improvement of confidence in caring for dementia patients’, helped me understand dementia patients through the experience of the most difficult mental behavioral symptoms in caring for dementia patients, and there were many opinions that it would be possible to apply the coping method to the aggressive behavior in dementia patients by returning to practice. This can be interpreted in the same context as the results of nursing students who performed virtual reality practical training in previous studies, which improved clinical performance and increased self-efficacy after practical training [30]. Furthermore, it can be seen in the same context as the result of increased knowledge compared to the group that received traditional lecture-style education as a result of conducting disaster preparedness education for nursing students through virtual reality. In addition, it can be interpreted in the same context as the result of reducing the stress of the subjects participating in the practice, in that it is possible to repeat the practice while experiencing a situation similar to actual clinical practice, and it is an experience that does not cause ethical problems or harms the patient [31,32,33]. In this way, the virtual reality practice program can be practiced with a sense of stability that it is safe to make a mistake, which can further increase the opportunity for practice through repeated learning and lead to improved confidence at work. 

The limitations of this study were ‘It was awkward and I was afraid to cover my eyes because it was my first experience’ and ‘I had dizziness while I was doing it.’ It can be seen in the same context as the research result that the need for a specific prior explanation of the virtual reality machine and mastery of the virtual reality machine is important [34,35].

In summarizing the results of the virtual reality program conducted in this study, the advantages were improved immersion in education and increased confidence, while the disadvantages were dizziness and discomfort in using the equipment.

Virtual reality programs are currently being used in everyday life and educational programs due to technological improvement, but they still have limitations through negative physical symptoms, such as nausea and motion sickness. This can be seen from the fact that in one study, 26 people experienced a virtual reality program using a TV screen and a mobile phone, and although the TV screen showed less motion sickness, both methods showed motion sickness [36]. This can be viewed in this same context, as the subjects in this study had dizziness symptoms. Accordingly, recent symptom measurement tools for virtual reality experience targeting virtual reality users have been developed. These include Virtual Reality-Induced Symptoms and Effects (VRISE), in which virtual reality participants can directly assess symptoms of their own experiences, and Virtual Reality Neuroscience Survey (Virtual Reality), in which symptoms can be measured in a shorter time. Further surveys include Neuroscience Questionnaire (VRNQ), Simulator Sickness Questionnaire (SSQ), and Fast Motion Sickness Score (FMS). Therefore, in the future, when conducting research using virtual reality, it is necessary to assess the physical symptoms of the subjects, and it will be necessary to consider ways to resolve them [37].

Considering measures to reduce these negative physical symptoms is a necessary measure at the present time, when the use of virtual reality programs is spreading. It also affects immersion, which is considered a big effect of virtual reality programs. Therefore, in order to realize the virtual reality program, it is necessary to analyze the problems that affect immersion. In a recent study that analyzed the ecology of immersion in virtual reality education in Korea using a grounded theoretic approach, unfamiliar user interfaces such as head-mounted displays and the recognition rate of machines (recognition of sounds and microphone recognition) were factors impeding immersion in virtual reality education. However, if these problems are solved, immersion will increase, and as a result subjects will distort the running time of the virtual reality program and feel it to be much shorter than the actual running time [38]. Recently, immersive VR has been developed overseas to increase the ecological validity of enhancing the positive experience of virtual reality. Immersive VR utilizes superior quality graphics, haptics and sounds, facilitates interaction with realistic 3D characters, and has ergonomic interactions and a VR-compatible navigation system. Therefore, it was found that subjects who experienced immersive VR did not show symptoms such as motion sickness, dizziness, and vomiting, which often appear after a VR experience [39].

Learning is a means to adapt to the environment, and in our daily life, we are constantly improving performance at work, improving academic achievement, and providing education to prevent dementia. Therefore, virtual reality education programs also need to integrate complexity, novelty, and diversity to provide an enjoyable experience to learners through the promotion of ecological relevance. This can enhance social interaction skills and cognitive ability through complex activities, such as music and sports that combine exercise and cognitive needs. Therefore, an ecological approach to the virtual reality education program can give learners a wide range of health, well-being, cognitive training, etc., so it is essential to consider this aspect in future virtual reality program development [40].

A review of this study based on the above related studies shows that the subjects of this study were all 65 years of age or older and were not familiar with virtual reality programs and devices. Therefore, in the future, it is thought that pre-education and preparation are necessary to be sufficiently familiar with the use of machines used for education before virtual reality education. Furthermore, in this study, scenarios and brief explanations of dementia were included in the virtual reality program, but the contents of these theoretical explanations may be factors that can enhance the negative physical symptoms, such as motion sickness by increasing the time of the virtual reality program. Therefore, it seems necessary to shorten the time for future program development, so that only the scenario can be experienced. In addition, it was judged that safety issues to prevent problems such as falls should be considered when involving older subjects. As such, in the development of virtual reality programs for education, it seems necessary to closely identify and develop problems in technological development that can reduce the negative physical experience of the subject, as well as prior education in order to increase the subject’s immersion.

## 6. Conclusions and Implication

This study analyzed the educational needs of the target, reflected the results, and developed and applied virtual reality practice for caregivers for the first time in Korea. However, there is a limit to generalize the effectiveness of this practice program because it was a pilot test as a result of analyzing the interview content conducted with only five caregivers currently taking care of dementia patients. We would like to suggest the results of this study as follows. First, caregivers are currently over 65 years old, and their experiences with virtual reality are limited. Therefore, since subjects were not familiar with the equipment used in virtual reality, it is thought that sufficient orientation and training on how to operate the equipment should be provided when conducting a virtual reality practice program for caregivers in the future. Second, this study was initially aimed at group education, but individual education was conducted due to the limited situation of COVID-19. In the educational needs analyzed in this study, the subjects also had opinions about information sharing through meetings with self-help groups of caregivers. In the future, it is possible to apply the method of sharing the psycho-behavioral symptoms of dementia patients experienced in clinical practice with each other, through group education, experiencing a virtual reality practice program, and sharing coping methods. This is thought to be an effective method through sharing various symptoms due to the nature of dementia and applying it to actual patient care by sharing various coping methods. Third, in addition to the virtual reality practice program developed in this study, it is necessary to develop various cases related to the psycho-behavioral symptoms of dementia patients, and repeated studies are needed to verify the effect with more caregivers. Lastly, this study provided a virtual reality education program only for caregivers who take care of dementia patients in long-term care facilities. However, in the future, these virtual reality education programs need expanded education not only for caregivers working in long-term care facilities, but also for family members and caregivers taking care of dementia patients at home. To this end, it is necessary to open such a virtual reality education program at the dementia safety center installed in each autonomous district, and to provide support so that any caregiver of dementia patients can experience and receive education. In the future, based on this study, it is necessary to develop scenarios for the psycho-behavioral symptoms of dementia patients in various themes tailored to the situation and environment. It will help to cope, and it will also help to reduce the burden on caregivers.

## Figures and Tables

**Table 1:** Analysis of domestic caregiver training courses.

Type	Subject	Education	Contents	Theory	Practical
Theory Lecture (80 h)/ Practical Practice (80 h)	Nursing Care Introduction	Nursing Care Related Systems and Services	- Social welfare system; - Overview of elderly health and welfare service system/visiting nursing; - Long-term care insurance service standards; - Resources related to health and welfare services for the elderly.	5	
		Purpose and Function of Nursing Care Work	- Purpose and function of nursing care services; - Basic principles and roles of nursing care professionals; - Type of nursing care service (facilities/home).	2	
		Caregiver Professional Ethics and Attitude	- Code of professional ethics and ethics in nursing care work; - Caregiver professional attitude; - Human rights of the elderly and prevention of abuse; - Self-management and safety management of caregivers.	8	6
		Understanding Nursing Care Recipients	- Elderly people characteristics (physiological and psychological characteristics); - Elderly people and family relationships.	2	
	Nursing Care Basic Knowledge	Medical Nursing Basic Knowledge	- Basic health conditions/major diseases of the elderly; - Health promotion and disease prevention of the elderly.	12	3
	Nursing Care Particular	Basic Nursing Care Technology(Ingestion, Excretion, Hygiene, Movement, Safety)	- Help with meals/help taking medicine and store medicine.	4	6
			- Help with use toilet/help with bed defecation; - Help with the use of a mobile toilet/diaper/infantile catheter.	5	8
			- Helping to clean the mouth, hair, hands and feet, and perineum; - Washing/helping with bathing/changing clothes/maintaining the environment.	5	8
			- Bed movement/wheelchair movement/walking/transportation assistance.	6	8
			- Fall/slip/tumble prevention; - Infection prevention and pressure sore prevention/aspiration.	3	6
		Housework and Daily LifeSupport	- Purpose, function, and basic principles of daily life support; - Meal preparation and nutrition management/food and tableware hygiene; - Cloth and bed cleaning and washing; - Assisting with going out and supporting daily work.	4	6
		Communication and Leisure Support	- Efficient communication/rapport-forming method; - Help with leisure activities (watching TV, listening to music, etc.).	5	6
		Service Support for Use	- Identification of characteristics of recipient and place and service plan; - Linkage with other occupations and other services; - Work report meeting, case review meeting.	3	4
		Nursing Care Work Record Report	- Purpose and importance of records and reports; - How to record/report work.	3	4
	Special Nursing Care Particular	Dementia Care Technology	- Daily life support/communication of dementia patients; - Coping with problem behaviors in dementia patients.	6	6
		End-Of-Life/Hospice Care	- Death and end-of-life/end-of-life care; - Hospice overview.	3	3
		First Aid Technology	- First aid (fracture, suffocation, convulsions, burns)/basic resuscitation.	4	6
Field Practice (80 h)	Elderly Care Facility Practice	Integrated Practice I		40	
	Home Care Service Practice	Integrated Practice II		40	

**Table 2:** Content analysis of the most difficult aspects of caring for dementia patients as a current caregiver.

Type	Category	Meaningful Content
Psychological symptoms	Delusion	I (patient) have to go find the man who had an affair with my wife (delusional jealousy of wife or husband). They (caregivers) told me to go on drugs (cups) to die quickly. Controversy over stealing a pension passbook and other passbooks.
	Suspicion	Stole my money. I (patient) suspect that if you(caregiver) open the closet to change clothes, you will search through them. I (patient) continue to doubt the caregiver, saying who took what every day. Items that are missing from the kitchen are often taken by the caregiver who sees them. Suspicion, often stating that things are lost (almost every day).
	Anxiety, Nervousness	Call every 2 min. Until I (patient) go to sleep, I try not to fall off, so I’m just afraid of being alone. When the patient screams for me to take him and follows me to the end.
Behavioral Symptom	Aggression	I (caregiver) feel mentally anxious and tired (suddenly attack). Physically dangerous, fearful when providing care. When yelling and holding a cane. Since there are only two patients and guardians in the house, there is no way to stop an attack (fear).
	Hallucination	Auditory hallucinations (imitation)/visual hallucinations: saying that things that are not constantly appear to exist. He (patient) beats the whole house with a long stick and weeps, saying that he can see the faces of his dead relatives. Talking to an invisible object and doing what the invisible object tells you. Shouting out saying somebody here to kill me. The act of screaming for help.
	Swearing/ Wandering	Cursed, but still hard to ignore the personality of the patient. Repeatedly using verbal violence and saying that he would be willing to kill. Same time wandering/hallucinations keep trying to get out. Behavior that disappears quickly (trying to get out).
	Repetitive behavior	Repeating the same question multiple times. Same words (inappropriate behavior) throughout 2 h of service every day. Repeat the same words every day (name).
	Etc.	Excretion: discard anywhere in the feces/touching feces with your hands. Refusal to bathe: refusal to take a bath even though the visible hands and feet are blackened. Sleep disorder: I (patient) can’t sleep at night and I wake up constantly (every 2 h) to urinate. Sexual behavior: embarrassed by rude jokes and actions.

**Table 3:** Content analysis of the most necessary education while caring for dementia patients.

Type	Category	Meaningful Content
Knowledge (Theory)	Dementia related knowledge	Without knowledge (theoretical education), mentally problematic behavior cannot be understood and properly performed. It is also necessary to understand hospital prescription drugs (dementia patients) (when I actually took care of them). Dementia specialist programs are considered necessary on a regular basis. I want the country to have a system in which all working people must receive dementia education. Understanding dementia patients (understanding dementia disease). Symptoms and characteristics of dementia patients by stage. Appropriate drug treatment. What are the psychiatric behaviors of dementia?
	Understanding dementia caregivers	Explaining the condition of the dementia patient to the caregiver. How to communicate with caregivers (how to deal with emotions). Talking and discussing care with the caregiver.
	Self-management education for caregivers with dementia	Self-management rather than knowledge (mental strengthening education to caregivers). Giving time to someone caring for someone with dementia (self-help group). How to share other people’s stories. How to deal with depression caused by caring for patients for a long time. How to manage sudden anger. How to be patient (requires a lot of patience). How to be professional, not compassionate.
Practice (Training)	Implementation of dementia prevention management program	Cognitive stimulation activity program to maintain and enhance cognitive function. How to play according to the condition and level of dementia patients. Information that slows the progression of dementia patients (play, activity). Rhythms or recreational movements that increase concentration or enjoyment. Exercise program to maintain residual function, nutrition management.
	Daily life care for dementia patients	Tips for changing diapers (difficult because the body is too big). Changing diapers when urinating, worrying about falls, difficult to do alone. Persuasion and understanding process for dementia patients (I want to see the process of persuasion actually). Bathing skills (rejection, risk of falls). Bedsore management, bath problems, meals, etc.
	How to take care of dementia patients	Caring for emergencies (response)/I am embarrassed and nervous about an emergency. How to deal with problem behavior (aggression, profanity). A variety of case-based education that can be used in case of emergency. How to respond when an elderly person finds a missing item. Due to the expansion of knowledge, it is necessary to conduct coping method education in theoretical education. Practical practice is required for each coping method according to the type of dementia. Skills to communicate with people with dementia.

**Table 4:** Virtual reality practice program for improving core practical competencies of caregivers.

Program Title	Korean-Style Virtual Reality Practice Program for Improving Core Practical Competencies of Caregivers			
Program Purpose	The purpose of this program is to lower the burden of caring for caregivers taking care of dementia patients, and to improve the quality of care for dementia patients by understanding aggressive behavior among the mental behavioral symptoms of dementia patients and performing coping methods.			
Program schedule	This program is a pilot test according to the development of a Korean-style virtual reality practice program for the improvement of core practical competencies of caregivers. The program will take about 70 min (60 min of program, 10 min of break time).			
Time	Topic	Educational goals	Contents	Method
10 min	Program introduction		Introduction of program schedule and progress. Pre-investigation (general characteristics, experience of caring for dementia patients).	Self introduction Pre-investigation
15 min	Understanding the psycho-behavioral symptoms of dementia patients	Explain the definition of psycho-behavioral symptoms in dementia patients.	Definition of psycho-behavioral symptoms in dementia patients. Types of psycho-behavioral symptoms in dementia patients.	Lecture
5 min	Conduct virtual reality practice	To understand the psycho-behavioral symptoms of dementia patients.	One of the mental behavioral symptoms of dementia patients, aggression due to hallucinations, can be directly experienced through virtual reality implementation practice from the perspective of dementia patients.	Lectures and Practice
10 min	Cases of mental behavior in dementia patients	Coping methods for psycho-behavioral symptoms of dementia patients can be performed.	Sharing of cases and experiences of psycho-behavioral symptoms that occur in actual caring of dementia patients.	Discussion
10 min	Coping with psycho-behavioral symptoms of dementia patients		Educating and sharing experiences on how to cope with the psycho-behavioral symptoms of dementia patients.	Discussion and Lecture
10 min	Program summary		Program satisfaction survey (survey, interview).	Post test Group interview

## Data Availability

Data from this study can be requested from the first author. The analyzed data is not disclosed to everyone, to protect the privacy and ethical protection of the subjects.

## Appendix A

**Scenario: Understanding and Coping Methods for Aggressive Behaviors Caused by Delusion in Patients with Dementia**

1. Topic setting and situation introduction.
  (1) Target: caregivers caring for dementia patients.
  (2) Subject: mental behavioral symptoms of dementia patients (aggressive behavior due to delusions).
  (3) Place of practice: researcher’s lab (Room 506, K University).
  (4) Learning Goals
    - Explain the definition of psycho-behavioral symptoms in dementia patients.
    - It is possible to understand aggressive behavior among the psycho-behavioral symptoms of dementia patients.
    - Among the psycho-behavioral symptoms of dementia patients, it is possible to explain how to cope with aggressive behavior.
  (5) Preparations: HMD (Head Mount Display), PC.
2. Situation Introduction

Choi 00, a 76-year-old man, has repeatedly forgotten words and interrupted conversations since a year and a half ago, and began to lose things such as wallets and cell phones. Due to the sudden death of his wife two years ago, he was living alone on a farm, and his children were all separated from him in the city.

Recently, he could not find his way and his children called the police station. When his children visited the police station, he was abusive and appeared violent. The eldest son visited the hospital with him and he was diagnosed with advanced Alzheimer’s. Afterwards, he was placed in a long-term care facility near his home.

After entering the long-term care facility, he said that due to the new environment and people, the caregivers who are currently taking care of him came to steal his money and tried to kidnap him, showing aggressive behavior. Among these behaviors, if the caregiver tries to take a bath for personal hygiene, he is very aggressive, trying to imprison himself and kill him. As a result, it is very difficult for caregivers to take care of him.

3. Virtual reality implementation image.
